# Combining Cognitive Mapping and indigenous knowledge to improve food environments in regional New Zealand

**DOI:** 10.1002/hpja.549

**Published:** 2021-10-27

**Authors:** Pippa McKelvie‐Sebileau, David Rees, Boyd Swinburn, Sarah Gerritsen, Erica D’Souza, David Tipene‐Leach

**Affiliations:** ^1^ Research and Innovation Centre Eastern Institute of Technology Napier New Zealand; ^2^ School of Population Health University of Auckland Auckland New Zealand; ^3^ Synergia Ltd Auckland New Zealand

**Keywords:** cognitive mapping, food, New Zealand, paediatric obesity, public health, schools, systems analysis

## Abstract

**Issue addressed:**

Hawke's Bay has one of the highest rates of childhood obesity in New Zealand. While several initiatives exist aiming to decrease obesity through physical activity, there are few nutritional interventions. This study adopted a systems science and mātauranga Māori approach to identify and target underlying drivers of rising childhood obesity and engage the community to improve the food environment.

**Methods:**

Cognitive mapping interviews (CM) with local stakeholders (school principals, Iwi and district health board representatives, education managers and local councillors) were conducted. The aim was to map participants’ mental models of the causes of rising childhood obesity and to identify key principles for engaging with the local community in a meaningful, impactful and culturally appropriate way for future action.

**Results:**

Eleven interviews were conducted face‐to‐face and cognitive maps were constructed. Follow‐up interviews were carried out online, due to COVID restrictions, to present the maps and for interviewees to make any adjustments. Four composite themes emerged through centrality and cluster analysis of the resulting cognitive maps: the importance of building in mātauranga Māori (Māori knowledge and ways of being), the “hauora” of children, working with the community and integrating existing initiatives. Two contextual factors are also considered: the growing need for food security in our communities and the opportunity to start interventions in the school setting.

**Conclusion:**

Cognitive mapping can produce useful insights in the early stages of community engagement. The six “pou” (pillars) underscore the importance of incorporating indigenous knowledge when embarking on public health interventions, particularly around obesity and in regional communities.

**So what?:**

When designing a public health initiative with a community with a high indigenous population, indigenous knowledge should be promoted to focus on holistic health, working with the community and creating opportunities for cohesion. These founding principles will be used to structure future community actions to improve children's food environments in regional New Zealand.

## 1 INTRODUCTION

Of 41 high‐income OECD and European Union countries, New Zealand has the second highest rate of childhood obesity, with 39% of children aged 5‐19 overweight or obese.^1^ In Australia and Canada, other countries with significant indigenous populations, this rate is 34% and 32% respectively.^1^ Children and young adolescents with overweight and obesity have significant health risks, a shorter life expectancy,^2^, ^3^ lowered self‐esteem and a psychological burden that can reduce participation in social life.^4^, ^5^, ^6^, ^7^ Patterns of obesity vary according to ethnicity and socio‐economic factors, with the most disadvantaged groups also experiencing food insecurity and irregular access to nutritious food.^8^

Regionally in New Zealand, childhood obesity rates show much variation, and in the Hawke's Bay (HB) region of New Zealand, one third (33.5%) of children are overweight or obese.^9^ A significant portion of the population identifies as indigenous, with 37% of all school‐aged children^10^ and 27.0% of the regional population identifying as Māori ethnicity, compared to the national average of 16.5%.^11^ There are proportionately more children in HB than in other regions, and high relative deprivation with just over half of the population living in the most deprived quintiles 4 and 5.^12^ Nutritionally, while HB is one of the largest fruit‐ and vegetable‐producing regions of New Zealand, in 2016/17, 29.0% of children did not meet fruit intake guidelines (2+ servings per day) and 58.3% did not meet vegetable intake guidelines (2‐3 servings per day, depending on age). While fruit intake is similar to the national average, this is the second lowest rate of vegetable intake per region in the country.^9^

The Nourishing Hawke's Bay: He wairua tō te kai initiative, was established in January 2020 as a collaboration between local public health researchers and a national University, funded by the National Science Challenge A Better Start. “He wairua tō te kai” is an adage adding cultural meaning, indicating that there is more to food than just nutrients. This study aims to engage the community in a shared vision for improved food environments in the region, recognising current barriers to systemic change, and to elicit community views on culturally acceptable and equitable options for a systemic intervention in the local food system. With the preponderance of Māori among children living with food insecurity in the region, we seek to usefully integrate mātauranga Māori (Māori knowledge base) into our approach to improving the regional food environment. This article focuses on the findings from phase one; cognitive mapping interviews (CM) with local stakeholders that define the issue(s) and orientate the ensuing interventions. Māori terms and concepts are used throughout this article and explained in Appendix A.

## 2 METHODS

A kaupapa Māori‐consistent framework was applied for all aspects of the research,^13^ respecting tikanga and mātauranga Māori (indigenous customs and knowledge), but recognising that the research team was a mix of indigenous Māori and tauiwi (of European descent). This values‐based approach is similar to that used by Tapera et al in a study on nutrition for children in a high deprivation area of NZ.^14^ We aimed to use a Māori worldview and deliver outcomes with Māori communities in mind.^15^

Insofar as possible, the CONSIDER statement^16^ for reporting health research involving indigenous peoples was respected in the design of the research and the preparation of this manuscript. A steering group for the research was established with representation from iwi, indigenous school principals and indigenous governance representatives from local councils. As these interviews were used to set the priorities for the ongoing research, high Māori representation at the stakeholder level ensured that the themes emerging would be of relevance to indigenous communities.

### 2.1 Ethics

The study was approved by the institutional Research Ethics and Approvals Committee (Ref 20/03). Interviews were audio‐recorded. The Standards for Reporting Qualitative Research (SRQR)^17^ have been followed to ensure complete, transparent reporting of this qualitative research study.

### 2.2 Participant selection

Purposive sampling was used to select participants from the researchers’ existing local networks across three sectors: health, local governance and education. Interviewees were selected based on their perceived ability to comment on wider community issues and to ensure a diverse range of views were collected. Because obesity is disproportionately experienced by tamariki and whānau Māori, specific efforts were made to ensure high Māori participation in the interviews and demanded a kaupapa Māori‐consistent approach to the research as described previously. We drew primarily from the connections of the lead Māori researcher who has long‐standing local links in primary care, public health and indigenous iwi‐based organisations, and from a range of low‐decile (high deprivation) school principals whose school rolls have high numbers of Māori students.

### 2.3 Cognitive mapping interviews

Procedure: The interviews took place face‐to‐face at the participant's place of work or at the research institution and were conducted by an experienced local Māori public health researcher and a doctoral candidate collaborating with a consultant with expertise in cognitive mapping and systems dynamics.^18^ Whanaungatanga including a short mihi (personal introduction of everyone present) and a karakia (prayer) were used to set the interview culturally, aligning with kaupapa Māori practices.

Two guiding questions were used in the interviews: *“If we wanted to run an initiative in HB to improve food environments for children, what would we need to take into account to make sure it was successful?”* This question was designed to elicit causal pathways from the deeper contextual society issues to the local context and current actions underway, as well as how the stakeholder or their organisation related to the issue of poor nutrition and regional childhood obesity. Using the cognitive mapping interview (CMI) technique described below, the main interviewer constantly sought clarification around ideas, using probing questions to identify the causes and consequences of various statements.

The second question asked: *“So, if we did take into account all of these factors we have just discussed, what would the impact be? What could we achieve?”* This question was designed to identify a shared vision for the project and the ways of working to achieve this.

Direct quotes from the interviews are used in this manuscript to provide examples and elaborate on the main findings.

### 2.4 Cognitive Mapping

Cognitive Mapping is based on Personal Construct Theory, which suggests that we make sense of the world in order to predict how the world will be in the future, and to decide how we might act or intervene in order to achieve what we prefer within that world.^19^ We used CM as described by Eden^20^ to capture stakeholders’ mental models. CM helps to reveal, the causal logic of the interviewee, visually describing their perspectives on the issue of concern, through a set of linked constructs that are drivers and consequences of the issue. CM is a tool used in systems research, which views issues, such as rising childhood obesity, as emerging from complex and adaptive systems.^21^

When constructing a cognitive map during an interview, the interviewer selects salient ideas from the interviewee's discourse as distinct “variables” or “constructs”.^22^ Relationships and hierarchies between the constructs are mapped as connecting arrows, indicating either a positive or a negative causal relationship (polarity). The general structure of the map places drivers of the issue at the bottom of the page and the “goals,” superordinate concepts, at the top. (See Figure 2 for an example.)

### 2.5 Mapping process

**FIGURE 1:**
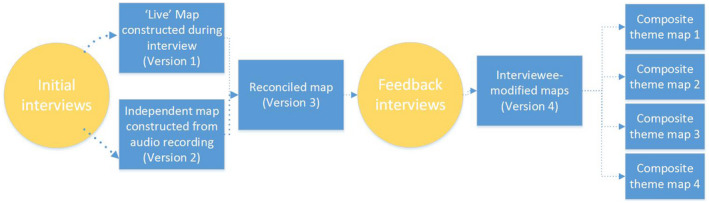
Cognitive Mapping Interview process and cognitive map versions

As illustrated in Figure 1, four versions of the individual maps from each interview were created through processes of validation and interviewee feedback.

*Version 1 ‐“Live” maps :* Construction of the cognitive maps began during the interview with one interviewer drawing the cognitive map and referring to it during the interview to check interpretation of comments.

*Version 2 ‐ Quality assurance / Independent map construction:* The full audio recording of the interviews was then analysed by a fourth researcher not present in the interviews and a second version of the maps was constructed using the Kumu© free online relationship mapping platform.

*Version 3 – Reconciled map:* The first author then reconciled Versions 1 and 2 to create a draft mental model map for feedback. Participants received this reconciled draft version of their individual cognitive map via email and were invited to participate in a short (20‐30 minutes) follow‐up interview to discuss and modify any elements of their map. In these follow‐up interviews, mostly conducted online via Zoom^TM^ due to COVID restrictions, we reiterated that the purpose of the map was to visually represent their ideas of the main drivers and causes of poor nutrition in the region, as well as identifying the consequences/impacts arising out of those issues. Participants were asked whether they thought the map reasonably captured their thinking and they were invited to modify the wording and relationships (eg Causal direction), or to delete constructs and/or causal arrows.

*Version 4 – Interviewee‐modified maps:* A final participant‐“validated” mental model map was created for each participant.

### 2.6 Composite/ thematic maps

Finally, composite theme maps were created using Banxsia, Decision Explorer Software to enable analysis and the construction of composite maps.^1^ To identify the “richness of meaning of each construct”^23^ from all 11 individual models, we used Decision Explorer for centrality analysis to identify the most densely connected constructs. That is, those constructs that have the most arrows going out, therefore a major driver, and those with most arrows coming in, that is, a key focal point and therefore a potential opportunity and/or barrier. Within cognitive mapping, constructs take their meaning from the context within which they sit, that is their causal relationships to the set of interconnected constructs with which they are linked. This allowed us to select the four principle constructs and to carry out a cluster analysis on each construct to look for the groups of related concepts (clusters). These central hubs formed the basis of the composite maps and all linked concepts from across the 11 individual maps were combined in the composite maps.

## 3 RESULTS

### 3.1 Interviewees and interviews

Eleven stakeholders were invited via email or via direct phone call (if they were well known to the authors) to participate in a 1‐hr face‐to‐face interview regarding food systems in Hawke's Bay and all accepted. Five were female and eight were Māori ethnicity. There were four low decile primary school principals, one local council member, one senior director in the local Ministry of Education, one public health physician, two district health board representatives and one iwi representative.

Interviews lasted on average 54 minutes (ranging from 45 minutes to 1 hour 13 minutes) and the maps constructed immediately following the interview (version 2) contained between 11 and 27 concept variables.

### 3.2 Characteristics of the cognitive maps

Across the original 11 individual maps, there were 374 concepts. While each map was different, representing an individual's perspective of the issue and their particular organisation's role, there were many areas of overlap between maps. Concepts referring to the same programme or initiative were merged to begin the process of identifying composite themes. For example, “Free and healthy school lunches” was merged with “Free school lunches.” Similar concepts were also merged, for example, “make sure community are involved from the start” and “talk to the community and involve them in problem solving” were merged as both referred to community involvement. Keyword searching was used to identify concepts referring to the same thing and as such, the original list of 374 concepts was refined iteratively to a list of 294 distinct concepts. Constructs were reworded for simplicity and interpretation as necessary, though all efforts were made to retain the original terms used by the interviewee.

### 3.3 Composite themes from the stakeholder interviews

**FIGURE 2:**
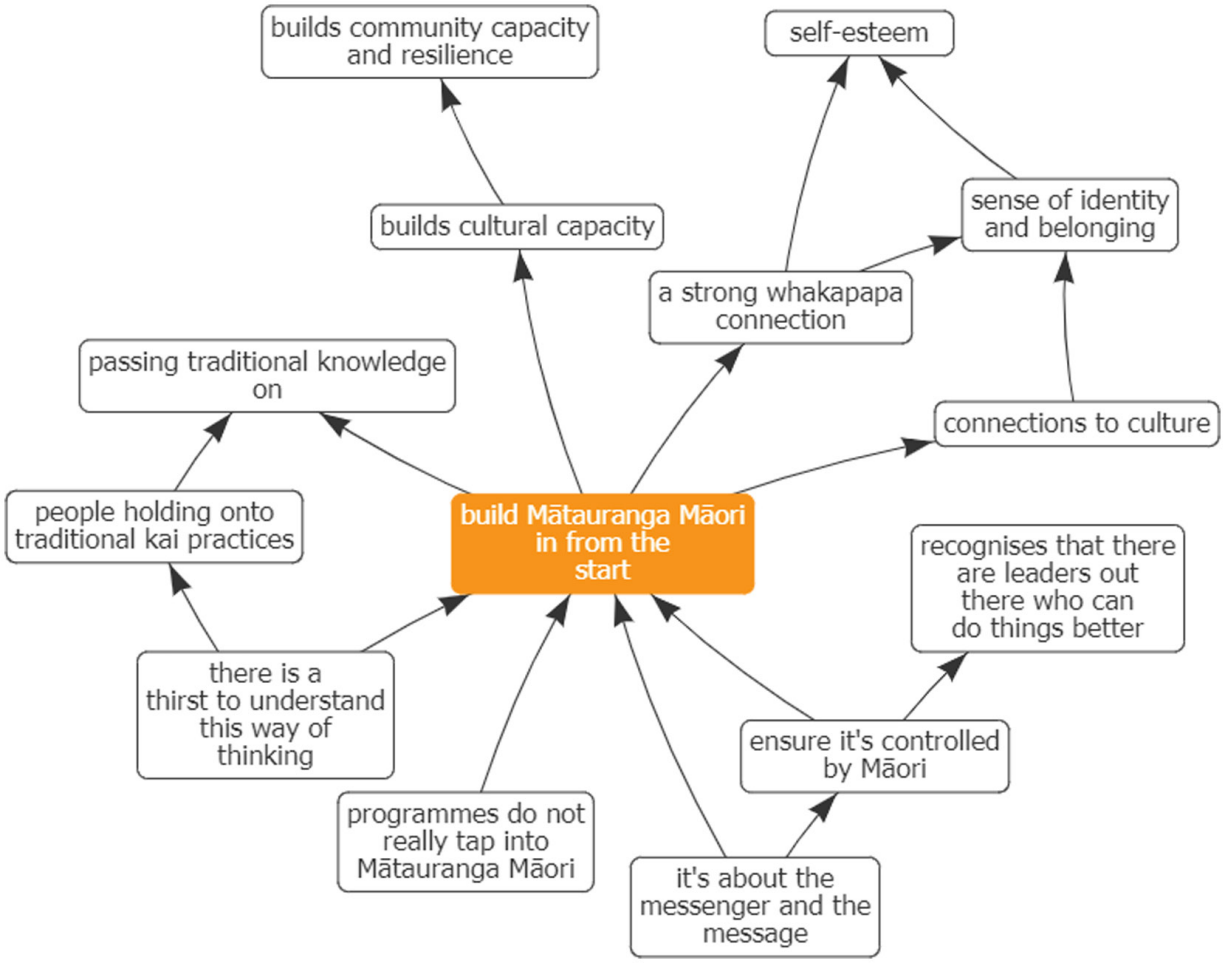
Composite Theme—Mātauranga Māori (Māori knowledge and belief systems)

**FIGURE 3:**
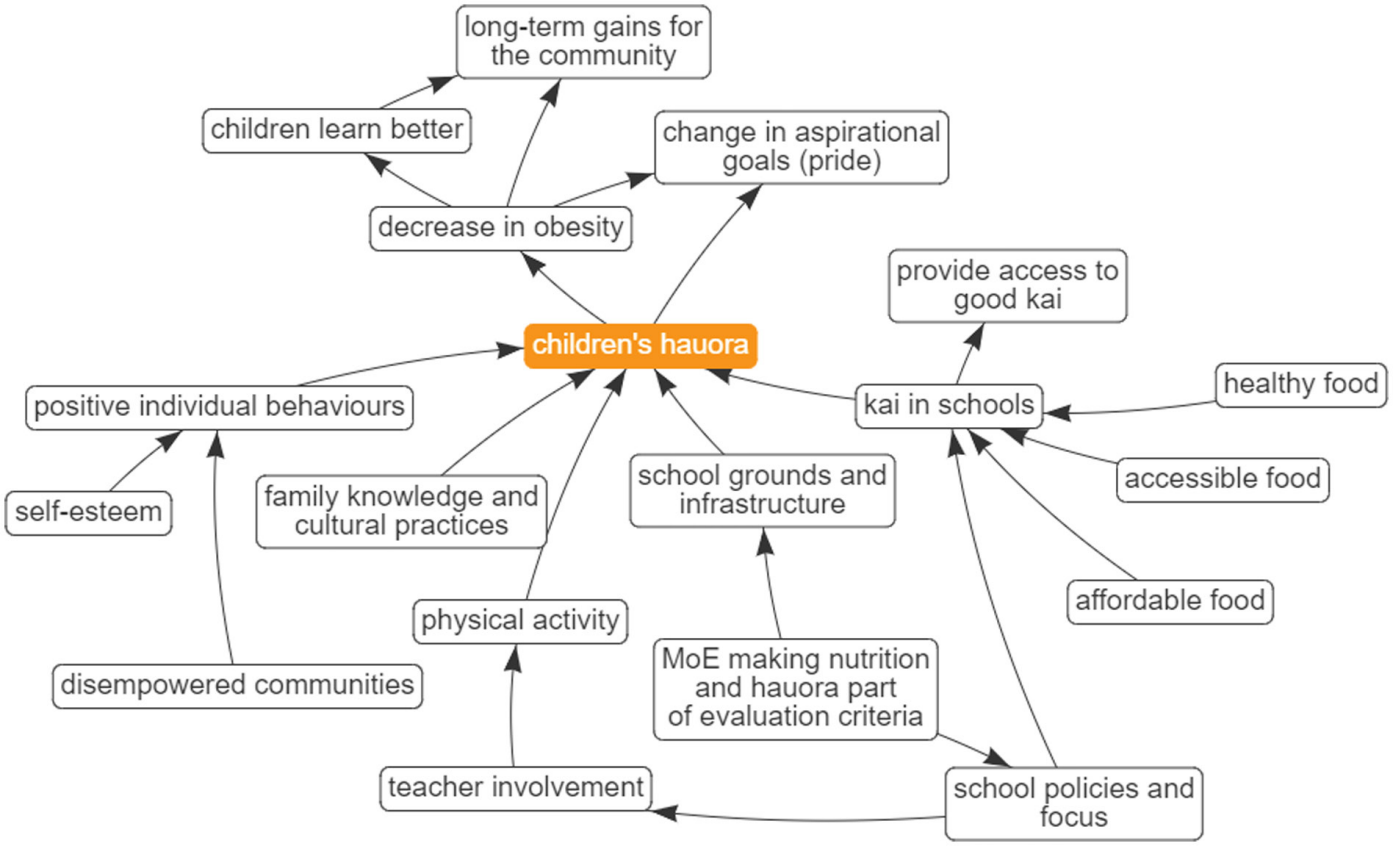
Composite Theme—Children's Hauora (Māori notion of holistic health taking into account multiple aspects of health)

**FIGURE 4:**
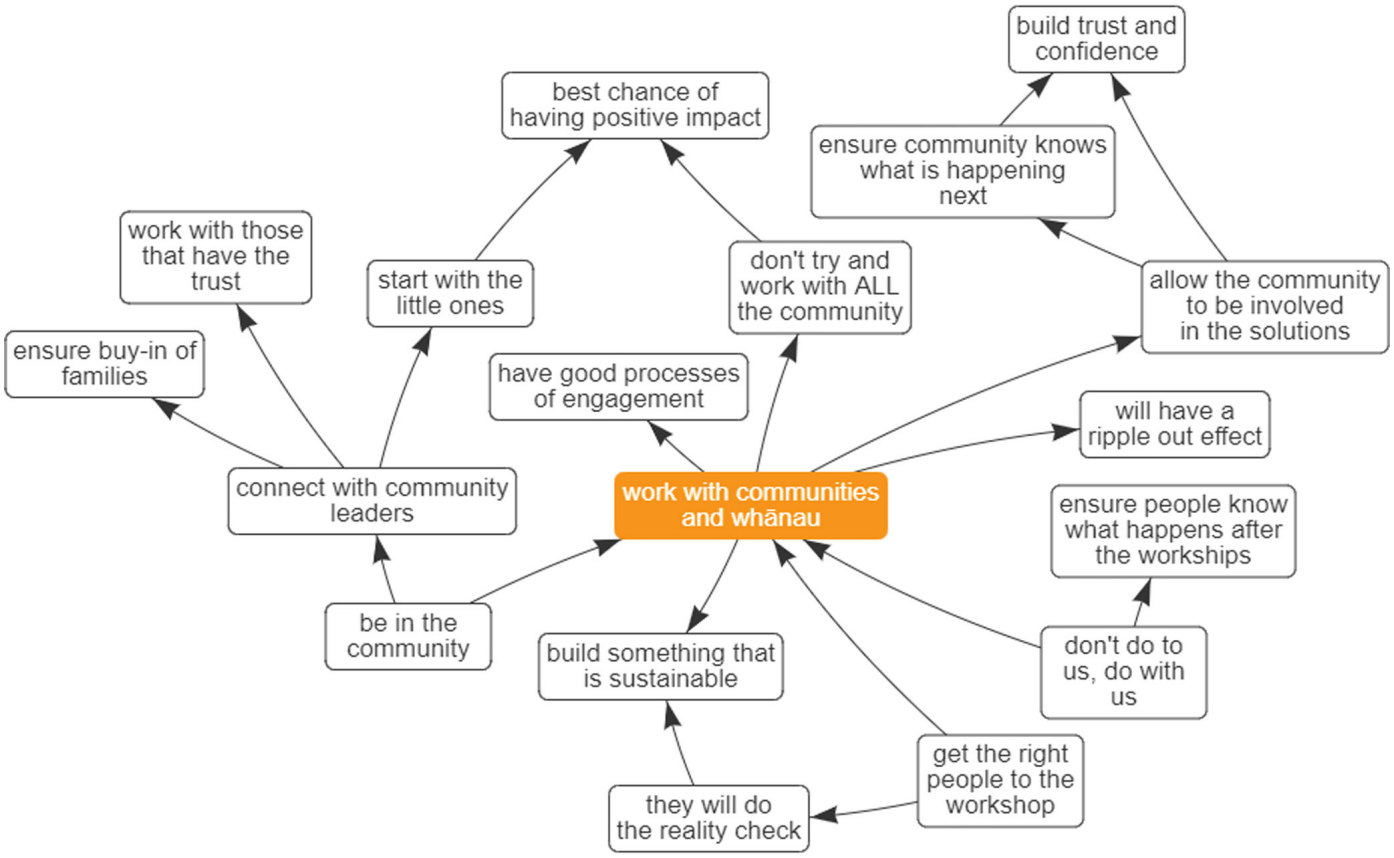
Composite Theme—Working with Communities and whānau

**FIGURE 5:**
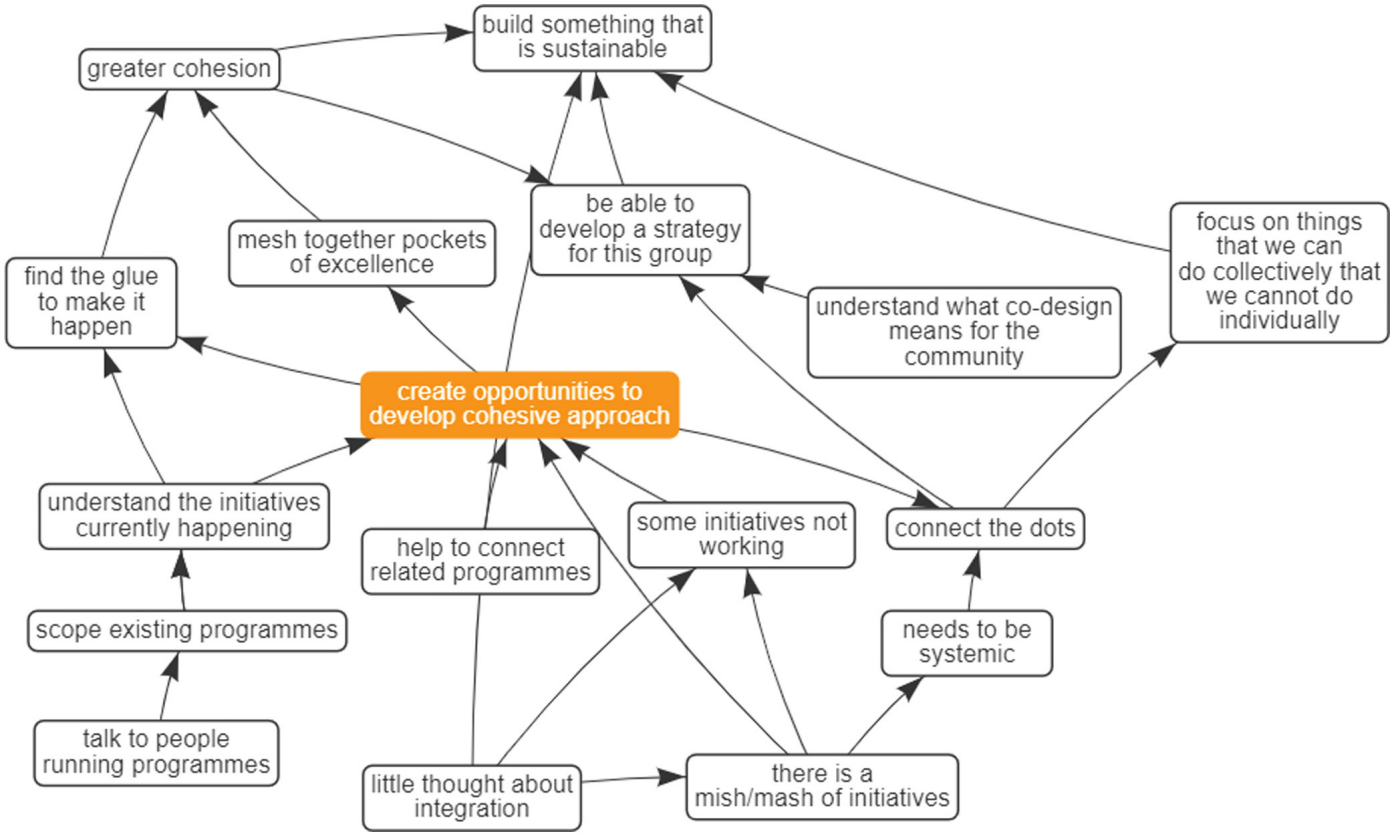
Composite Theme—Creating Cohesions

Four key themes or foci were identified through the process of refining the maps and analysing the central hubs. These themes represent ways of working that held high importance for the interviewees and as such, we consider these to be the foundational pillars or principles for the wider project. The concepts, which will be discussed in detail below were: Building in Mātauranga Māori (Māori knowledge and belief systems) right from the start, considering Children's Hauora (holistic health), Cohesion & Integration, and Working with Community (Figures 2, 3, 4, 5).

Mātauranga Māori (Figure 2) takes into account indigenous Māori knowledge, culture, values and world view. Specifically, it includes the knowledge forms of pūrākau (creation mythology) and maramataka (moon cycles) and can be considered to incorporate “knowledge generated using techniques consistent with the scientific method, but explained according to a Māori world view”.^24^ In this composite map, we see that building mātauranga Māori into a public health intervention was seen to be a means of connecting individuals and communities with their culture, through reinforced whakapapa links and giving individuals a sense of identity and belonging. Building in Mātauranga Māori was perceived as an appropriate way to build community capacity and resilience to break the feedback loop of unhealthy food environments.

Hauora (Figure 3) is a broad term, generally used to refer to a Māori philosophy of wellbeing that offers a more holistic view of (an individual's) health than a Western scientific approach. It is often translated as, or juxtapositioned with, “wellbeing.” The terms “hauora” or “wellbeing,” came up in six different constructs across individual maps. In this composite map, we see the various mechanisms by which hauora could be enhanced. For example, participants mentioned that if the correct governance structures were in place to ensure that schools had nutritional and physical activity policies, with the Ministry of Education assessing schools on whether these were in place and operational, this could immediately improve children's hauora in a school. Kai (food) in schools was another major subtheme to come through. It was seen as both a means to ensure food security with regular access to nutritious food, but also as a way of introducing healthier food, exposing children to healthier food and different tastes earlier, and in turn enhancing hauora. In addition to the physical benefits of a decrease in obesity, enhancing hauora and wellbeing were seen to be educational (better educational outcomes), and also societal, with a change in community aspirations and long‐term community benefits.

All stakeholder interviewees emphasised the necessity of working with Community and whānau (families and extended families) (Figure 4). A strong sub‐theme emerging underscoring the ethics and reciprocal obligations of the researcher when working with a community. Interviewees spoke of the need to ensure that people (including participants and wider community) know what is happening in the project and that participants do not feel that they are “subjects of” the research. Through good processes of engagement and ensuring that the community is involved in co‐designing the solutions, the initiative would gain the trust and confidence of the community to effect sustainable change. These ideas align with kaupapa Māori research principles.^13^ By embedding the actions within a community, the initiative could have a ripple‐out effect. Specific concepts were to work with community leaders and to spend time thinking about inviting the right people to the future community group model building workshops.

The fourth major theme identified was the need and opportunity to Create Cohesions (Figure 5) to join people, projects and institutions together. Interviewees felt that there was currently a number of initiatives in the health improvement sector in the region, but that not all were working, and not all were talking to each other. They saw the need for a successful project to connect the dots and bring related programmes together. If our initiative managed to achieve this, we would enhance cohesion and build something that could offer long‐term benefits. This is exemplified in the quote to “focus on things that we can do collectively, that we cannot do individually.”

### 3.4 Additional contextual factors

Overall, the main recurring theme regarding the drivers of rising childhood obesity was the impact of growing inequities in the region and increasing poverty for families with young children. With particular regard to the focus of the Nourishing HB project, food security was identified as an issue with a serious impact on the children's health, and low decile primary schools were seen as a key location to intervene to facilitate regular access to healthier food for children.

*“There’s too much poverty. People are in emergency housing for example, in motels. How can you cook healthy food for your kids if you live in a motel with no kitchen?” [Programme Manager]*

*“In about the second week I was here, one of our girls turned up with a 2L ice cream for breakfast. I said “What are you doing girl?” and she said “Oh I can’t find a pie”. I thought: This has to change.” [School principal, low decile primary school]*.

One of the school principals spoke of the efforts in their low decile primary school to meet the needs of hungry children. In this particular school, a barbecue lunch for the students and families was provided on Fridays, to “fill their bellies, as we realised they weren't being fed over the weekend.” While the principals were able to give first‐ hand accounts of observing children without regular access to food and the impacts on their lives and learning, government stakeholders also mentioned the impact of poverty—stating “how can nutrition even get on the radar when there are so many other issues things [like gangs, violence and meth] going on in whānau lives?” Another low decile principal mentioned that being concerned about healthy food was like being concerned about climate change—“a problem for rich people with no other more pressing issues in their lives.” Starting food security and nutrition interventions in schools was seen as an important opportunity to level the playing field for students irrespective of their household environments. These two factors were added as guiding principles, “pou” for the overall project approach.

## 4 DISCUSSION

In this paper, we detail how cognitive mapping interviews deployed in a predominantly Māori, high deprivation community and school context were used to elicit the perspectives of local stakeholders from a range of organisations around the drivers of rising childhood obesity and poor nutrition in regional New Zealand. The eleven interviewees each had unique mental models of the causes of this public health issue, from disempowered communities to lack of nutritional knowledge for families, to screen use and low rates of physical activity. Particular mention was made in several interviews of the ubiquity of food insecurity in the region, and that schools were seen as a key location for increasing access to healthy food for communities where poverty prevented access to nutritional kai.

Drawing the cognitive maps while interviewing participants proved an effective way of exploring interviewee's causal logic and engaging interviewees. As discussed by Black,^25^ this shared visual presentation played a role in developing a shared understanding, to assist interviewees to understand the links and relationships between the constructs of the underlying food system; to “think with what they see.” It also maintained a connection and explained the interpretation and use of their contributions in an open, transparent manner. This was particularly timely for the project as engagement with stakeholders was important to establish relationships before the group model building workshops and lay the foundations for a successful intervention.

Across the maps, we identified key similarities and four major themes. These composite themes were formulated into composite cognitive maps, drawing in constructs from each of the eleven individual maps. With the addition of two contextual factors, this established the six guiding principles, which we called the “Pou.” In mātauranga Māori, pou can refer to physical pillars supporting the roof of the whare (meeting house), metaphoric columns (people) that are strong supporters of a cause, or a territorial symbol, such as a mountain or landmark, representing that support. In this context, we use them to represent the founding principles for the ways of working for the wider Nourishing HB: He wairua tō te kai project.

**FIGURE 6:**
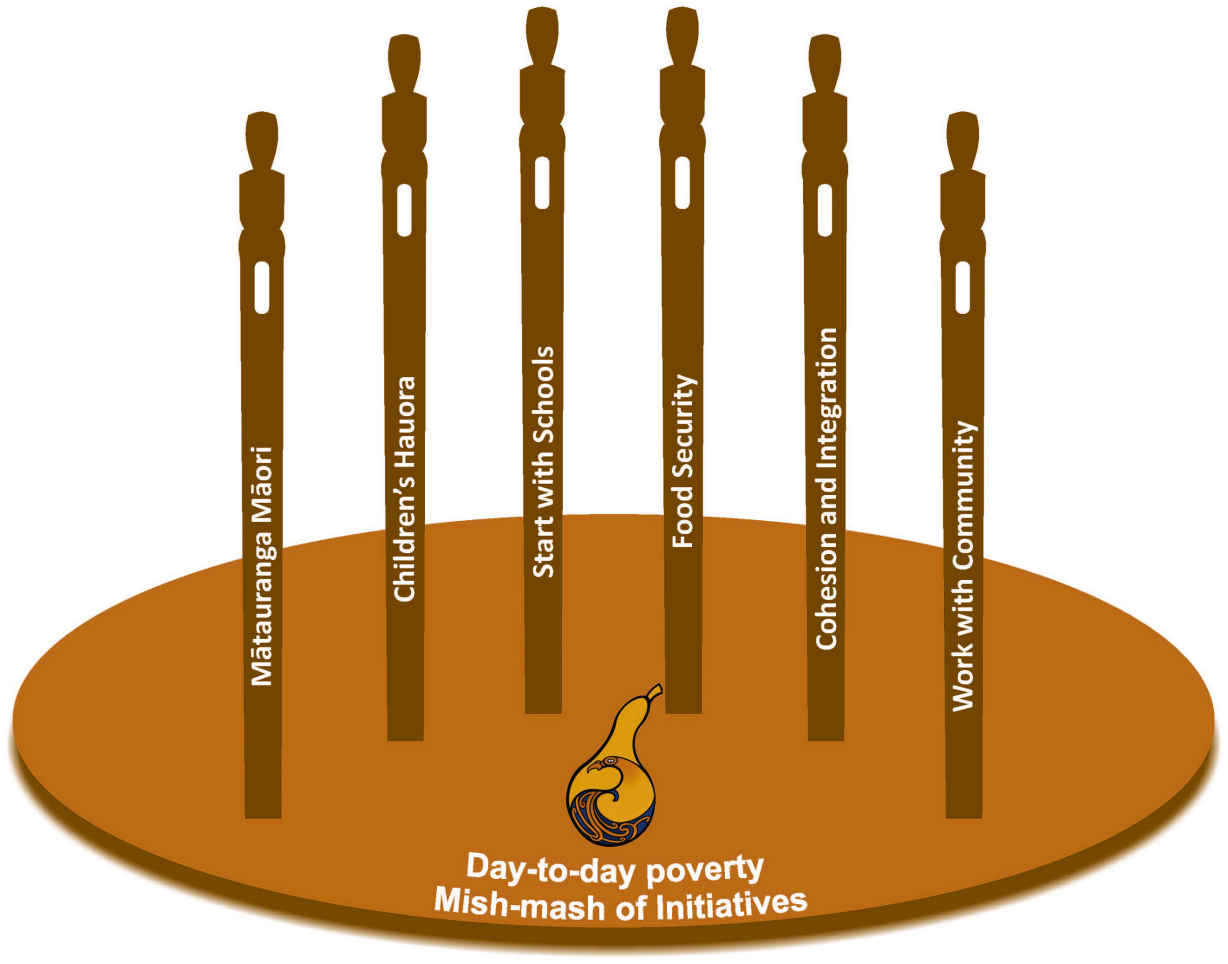
Six pou (pillars) of the Nourishing Hawke's Bay: He wairua tō te kai systems approach established by the cognitive mapping interviews

The six pou presented in Figure 6 provide the following foci:

- Building in Mātauranga Māori: building upon and recognising the importance of mātauranga Māori to provide knowledge from outside of the traditional western science paradigms that can empower individuals, both Māori and Pākeha, and communities to change systems;
- Children's Hauora: taking into account a holistic view of health as a key outcome measure, in particular in schools;
- Working with Community and Whānau: working with the local community in an ethical and responsible way to involve them in the design of the initiative, the selection of solutions and inform them of progress;
- Integration and Creating Cohesions: developing a cohesive approach to connect people, projects and initiatives so that collective action can be achieved;
- Taking into account the reality of food insecurity across the community and working to alleviate this before focussing in on nutrition; and
- Using the school setting as an opportunity to provide nutritious food and nutrition education to level the playing field for students irrespective of household environments.

Inequity in Māori communities was an underlying ethos for this project. Utilising Māori networks was the key to eliciting and maintaining participation. Not surprisingly, Mātauranga Māori emerged as an integral aspect to incorporate into any approach in public health, both to ensure that the initiatives connect with underlying values and framework of empowerment for communities, as well as a means of connecting with whakapapa (cultural identity and community networks) and increasing a sense of identity and belonging within a cultural space. Since the integration of Vision Mātauranga in 2010, a NZ government initiative to unlock the science and innovation potential of Māori knowledge, people and resources, mātauranga Māori has gained recognition and importance in mainstream institutions. The working with the community pou and reciprocal responsibility of researchers resonates strongly with kaupapa Māori research principles.^13^

Placing Mātauranga Māori at the forefront of community based initiatives has also been highlighted by Harding et al^26^ as a core component of effective co‐designed initiatives to address health inequities. This is both to promote Māori knowledge and ways of being as well as reducing the dominance of Western colonial methods.^27^ Furthermore, utilising indigenous knowledge was seen as a “strengths‐based” method, as opposed to deficit focussed analysis of inequities in indigenous communities which were not considered successful ways of engaging indigenous communities. This is echoed in recent work by Tonumaipe'a et al^28^ who seeks to change the metaphors utilised around food environments, to move from “food swamps,” to “food havens.” In a recent study working with Aboriginal communities in Australia, Browne et al^29^ found that Group Model Building, a related research methodology from systems dynamics, was a good fit with an aboriginal worldview and could build capacity within aboriginal communities address previous weaknesses of research on, rather than with, Aboriginal communities.

The importance and prevalence of irregular access to nutritious food was an unanticipated finding in the interviews but this reflects the growing inequities in NZ.^30^ The COVID‐19 lockdown has exacerbated these inequities,^31^ with data being reported in NZ on widening inequities in manifold areas from school attendance, job security and an estimate 50% increase in use of foodbanks.^32^ Gerritsen et al^33^ work using CM to map causal pathways for declining fruit and vegetable intake in the Auckland region also found barriers to fruit and vegetable intake to include poverty and high food prices, as well as low skills/knowledge, unhealthy food environments, climate change and urbanization.

It became very clear that one potential means of improving hauora and access to healthier kai for children from higher need communities, was through their schools. A number of disjointed initiatives were already underway, as well as a few schools who were participating in the first wave of the government funded free school lunches programme, and these were all seen as promising ways to break cycles of food insecurity.

Some limitations should be considered when interpreting these results, this relatively small group of participants were selected purposively mostly from existing networks and may not include the views of the full community. The regional setting should also be considered, although arguably, the findings about the importance of mātauranga Māori are applicable nationally under the premises of Te Tiriti o Waitangi, and indigenous knowledge has been recognised as necessary part of public health interventions globally.

## 5 CONCLUSIONS

In this series of interviews with local stakeholders in the food environment in regional New Zealand, we used cognitive mapping to elicit perspectives on the rising childhood obesity in the region and what a successful initiative would need to consider to address this. Four composite themes emerged through centrality and cluster analysis: the importance of Mātauranga Māori in public health interventions; focussing on children's hauora; working with the community; and integrating initiatives. These themes became key “pou” (pillars) around which the Nourishing Hawke's Bay: He wairua tō te kai community group model building workshops could be established. The cognitive mapping interview method was an effective way to elicit stakeholders’ views on the central “nubs” of concern and focus, in of the community's food environments. When designing a public health initiative with a community with a high indigenous population, indigenous knowledge should be promoted to focus on holistic health, working with the community and creating opportunities for cohesion. These four founding principles will be used to structure future community action to improve children's food environments in regional New Zealand.

## CONFLICTS OF INTEREST

The authors declare no conflicts of interest.

## ETHICS APPROVAL

This study obtained ethical approval from the Eastern Institute of Technology Research and Ethics Approvals Committee, ref 20/03.

## ENDNOTE

^1^

https://banxia.com/products/

## APPENDIX A Māori lexicon utilised in the manuscript. Definitions adapted from Te Aka Online Māori Dictionary

**Table:**

Māori term	Explanation
hauora	A general term for all aspects of health, encompassing physical, mental and spiritual health (for example, see Te Whare tapa whā described in the text). Generally utilised to communicate a wholistic view of good health and vigour.
iwi	Kinship group or ‘tribe’
kai	food
kaupapa	topic or initiative. (Kaupapa Māori‐consistent)
mahinga kai	Traditional practice of gathering food like kaimoana (seafood)
maramataka	Moon cycles and knowledge
Mātauranga	Theoretical or practical knowledge acquired through education and/or experience.
pou	Pillars
pūrakau	Creation mythology / legends
tamariki	children
te Ao Māori	Māori world(view)
tikanga	Correct procedures, custom, habits, conventions and protocols of te Ao Māori. The customary system of values and practices that have developed over time and are deeply embedded in the social context.
wairua	While wairua is for the soul or spirit of a person, in the sense utilised here for an inanimate object (kai), we mean the quintessence, nature or essence of an object.
whakapapa	The genealogical descent of all things including the Ātua (gods) and natural phenomenon like mountains and rivers. Whakapapa/genealogy is a fundamental principle that permeates the whole of Māori culture. [Kinship, genealogy, lineage and descent].
whānau	Family; Fundamental unit of kinship (followed by the larger hāpu and iwi). Generally utilised to include a broad definition of extended family including several generations of relatives.
